# Supplementary material for: Observation of anomalous amplitude modes in the kagome metal CsV3Sb5
Source: Nat Commun. 2022 Jun 16;13:3461. doi: 10.1038/s41467-022-31162-1 (PMC9203454; doi:10.1038/s41467-022-31162-1)
Supplement: Supplementary file 1 — Supplementary Information [file 41467_2022_31162_MOESM1_ESM.pdf]

Supplementary Information:
Observation of anomalous amplitude modes in the kagome metal CsV_3Sb_5

Gan Liu, Xinran Ma, Kuanyu He, Qing Li, Hengxin Tan, Yizhou Liu,
Jie Xu, Wenna Tang, Kenji Watanabe, Takashi Taniguchi, Libo Gao,
Yaomin Dai, Hai-Hu Wen, Binghai Yan,* and Xiaoxiang Xi†
*binghai.yan@weizmann.ac.il, †xxi@nju.edu.cn

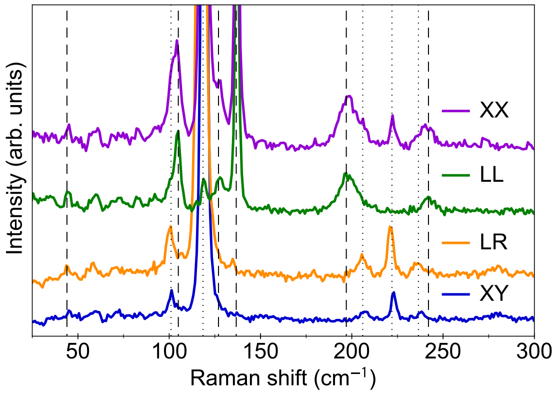

Supplementary Figure 1. Polarization resolved Raman spectra of CsV_3Sb_5, measured at 4 K. The dashed and dotted lines mark modes with A_{1g} and E_{2g} symmetries, respectively.

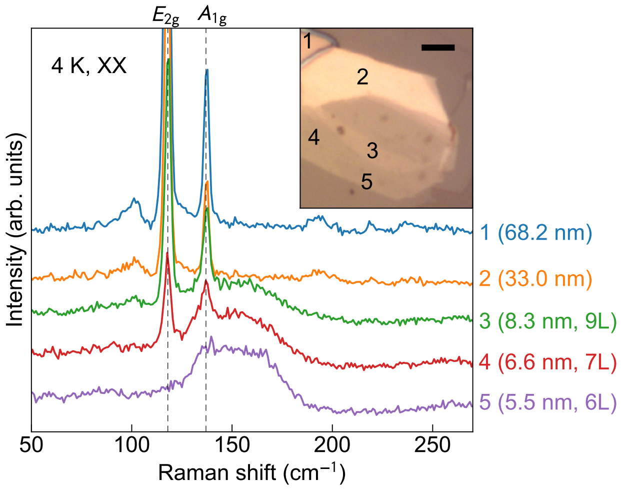

Supplementary Figure 2. Thickness dependent Raman spectra of CsV_3Sb_5 measured under the same conditions, vertically shifted for clarity. The inset is the image of the sample encapsulated by thin h-BN. Scale bar: 5 μm. Sample thickness was measured using atomic force microscopy. Thickness reduction leads to loss of crystallinity, as evidenced by the disappearance of the E_{2g} and A_{1g} modes. Sample preparation either in high-purity nitrogen or argon gas yielded similar results.

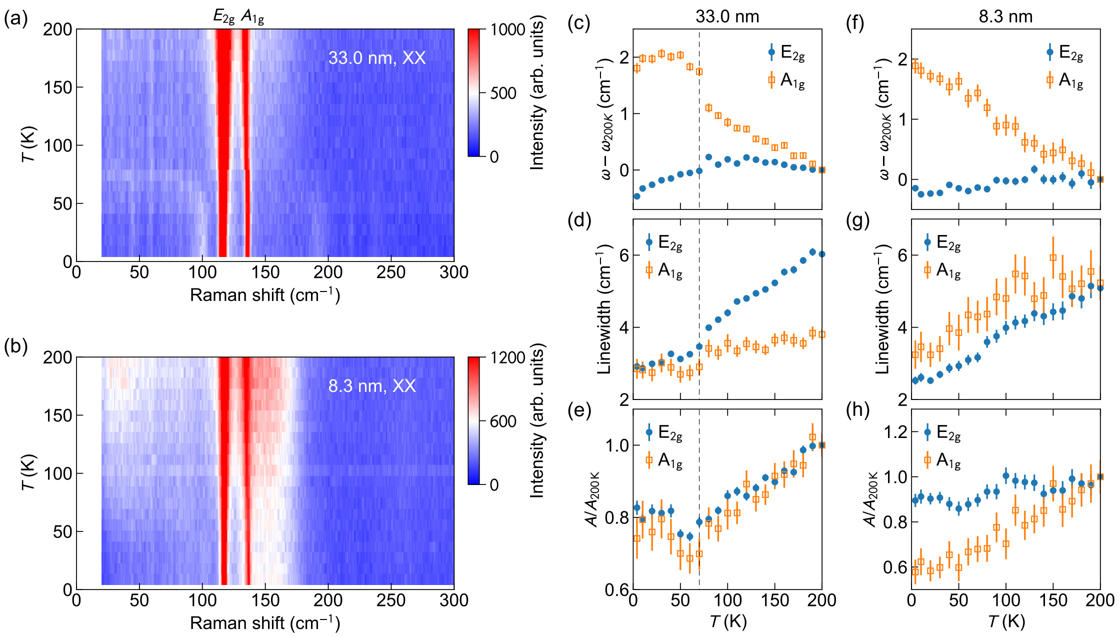

Supplementary Figure 3. (a, b) Temperature-dependent Raman intensity color plot for CsV_3Sb_5 measured for the (a) 33.0 nm and (b) 8.3 nm regions in Supplementary Figure 2. (c–e) Frequency, linewidth, and amplitude for the E_{2g} and A_{1g} main lattice phonons for the 33.0 nm region. (f–h) The corresponding data for the 8.3 nm region. T_{CDW} is suppressed to about 70 K in the 33.0 nm region, as indicated by the dashed lines in (c–e). No CDW transition is detected in the 8.3 nm region, as evidenced by the lack of anomalies in the phonon parameters in (f–h). Error bars are standard deviations obtained from the least-squares fits to the phonon peaks.

Supplementary Table 1. Frequency (in cm^{-1}) of the Raman-active modes in the pristine and $2 \times 2 \times 1$ ISD phases of CsV_3Sb_5. DFT: calculated. Expt.: measured at 4 K.

Pristine		A_{1g}	E_{2g}				
	DFT	136.7	128.6				
CDW		A_1	A_2	A_3	A_4	A_5	A_{1g}
	DFT	–	108.1	131.5	202.9	241.9	141.5
	Expt.	44.6	104.0	127.2	197.4	241.5	136.7
		E_1	E_2	E_3	E_4	E_{2g}	
	DFT	99.0	212.2	221.0	233.6	129.0	
	Expt.	101.5	205.9	222.0	237.2	118.6	
		E'_1	E'_2	E'_3			
	DFT	57.5	124.8	175.2			

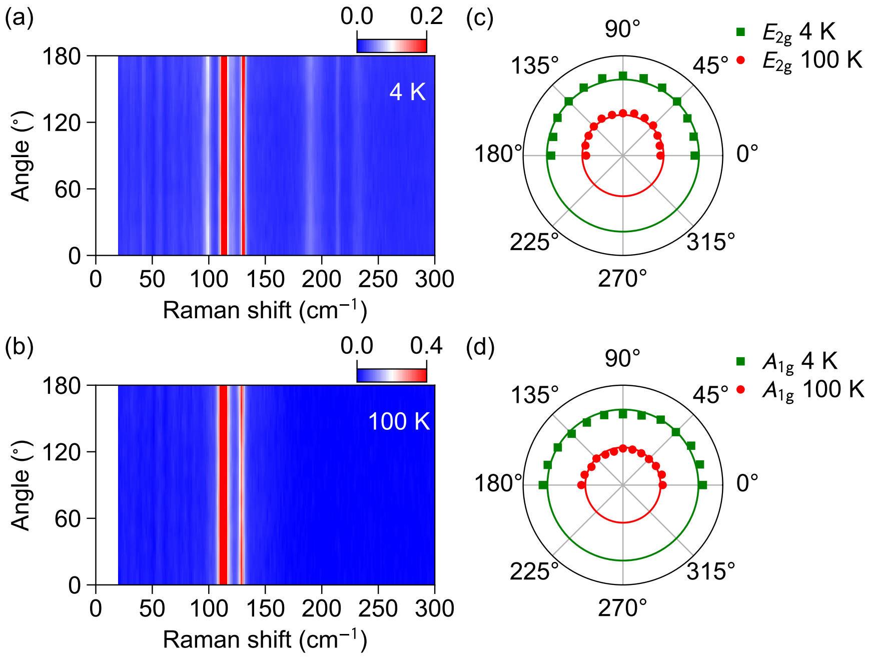

Supplementary Figure 4. (a) and (b) Polarization angle dependent Raman intensity color plots for the CDW and normal phases, taken at 4 K and 100 K, respectively, in the collinear polarization configuration. (c) and (d) Polarization angle dependence of the intensity of the main lattice modes. The symbols are analyzed values from Lorentzian peak fitting. The circles represent average values of the data points. Small variations are present due to experimental uncertainties. The standard deviation of the analyzed angle-dependent intensity divided by the average value yields the following results: 4.0% (E_{2g}, 4 K), 5.5% (E_{2g}, 100 K), 4.3% (A_{1g}, 4 K), and 5.3% (A_{1g}, 100 K). The slightly lower values at 4 K can be attributed to enhanced signal-to-noise ratio due to the strengthening of the main lattice phonon peaks, which do not support CDW-induced D_{2h} point group.

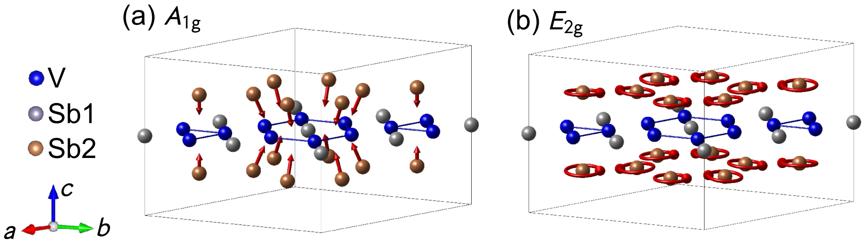

Supplementary Figure 5. Displacement patterns of the main lattice phonons in the $2 \times 2 \times 1$ ISD phase. (a) The A_{1g} mode. (b) The E_{2g} mode. The Cs atoms are omitted in the crystal structure.

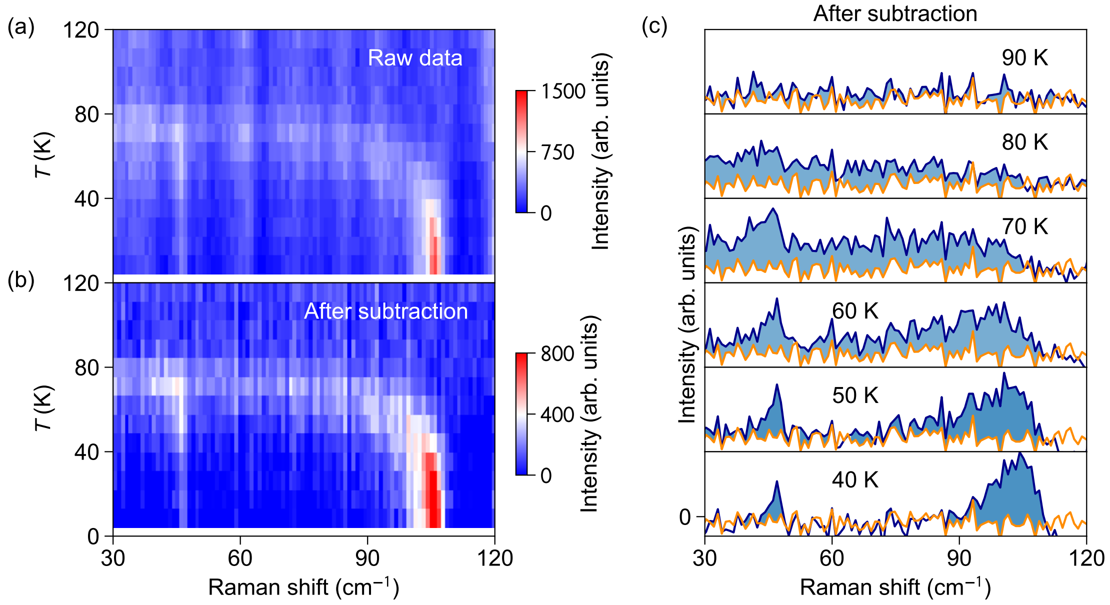

Supplementary Figure 6. A detailed inspection of the A_2 mode. The Raman intensity color plot of (a) the raw data and (b) the data after subtracting the 130 K spectrum. (c) Spectra at 40–90 K (blue) and 120 K (orange) after subtracting the 130 K spectrum. The shaded blue highlights CDW-induced intensity, mainly from the A_2 mode that redshifts and broadens upon warming, but also from the A_1 mode at 45 cm^{-1} which does not shift with temperature.

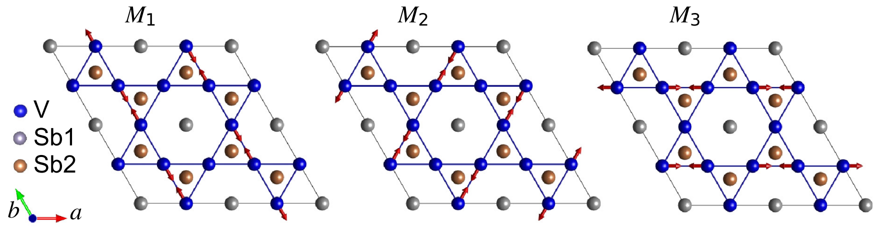

Supplementary Figure 7. Imaginary phonon modes of pristine CsV_3Sb_5 at three M points, $M_{1,2,3}$. Because the phonon dynamical matrix eigenvector has a π phase at M, we plot it in a 2×2 supercell.

Supplementary Note 1: Chiral character of E_{2g} modes

For E_{2g}, the doubly degenerate modes correspond to states with opposite angular momentum ($l = \pm 1$), i.e. they are chiral. Although the dynamical matrix eigenvectors are usually exported as real vectors by suppressing l, we can reconstruct the chiral phonons by a projection operation to chiral states. For C_3, the projection operator is defined as:

$$P_l = \sum_{k=0}^{2} (q_l^* C_3)^k.$$

$q_l = e^{i\frac{2l\pi}{3}}$ is the eigenvalue of C_3 with pseudoangular momentum $l = -1,\ 0,\ 1$. For an arbitrary phonon dynamical matrix eigenvector $\mathbf{u}$, the projected eigenvector $P_l\mathbf{u}$ must be chiral, because

$$\begin{aligned} C_3 P_l = C_3 \sum_k (q_l^* C_3)^k &= \sum_k (q_l^*)^k C_3^{k+1} = \sum_k (q_l^*)^{k-1} C_3^k \\ &= (q_l^*)^{-1} \sum_k (q_l^* C_3)^k = q_l P_l, \end{aligned}$$

i.e. $C_3(P_l\mathbf{u}) = q_l(P_l\mathbf{u})$.

Supplementary Note 2: c-axis modulation

Below we consider Raman-active modes for different forms of c-axis modulation. At Γ point all phonon modes can be classified into acoustic and optic modes, i.e. $\Gamma_{\text{total}} = \Gamma_{\text{acoustic}} \oplus \Gamma_{\text{optic}}$. The optic phonon modes can be further classified into infra-red (IR) active, Raman active, and silent modes as $\Gamma_{\text{optic}} = \Gamma_{\text{IR}} \oplus \Gamma_{\text{Raman}} \oplus \Gamma_{\text{silent}}$. Based on group theory analysis [Kroumova et. al. Phase Transitions, 76, 155 (2003)], we have derived the symmetry representations of phonon modes for five different modulations along the c axis:

(i) $2\times2\times1$ CDW composed of inverse star of david (ISD) structures with no modulation along the c axis (Supplementary Tab. 2),

(ii) $2\times2\times2$ CDW composed of ISD structures having an interlayer π phase shift (Supplementary Tab. 3),

(iii) $2\times2\times2$ CDW composed of ISD and SD structures having no phase shift (Supplementary Tab. 4),

(iv) $2\times2\times2$ CDW composed of ISD and SD structures having π-phase shift (Supplementary Tab. 5),

(v) $2\times2\times4$ CDW composed of one ISD and three consecutive layers of SD structures having no phase shift (Supplementary Tab. 6).

These structures either have the D_{6h} or D_{2h} point groups. For the former (latter), the Raman-active modes that can be observed in the back-scattering geometry of our experiment

are the A_{1g} and E_{2g} (A_g and B_{1g}) modes. Except for the $2 \times 2 \times 1$ structure, the predicted number of modes far exceeds that observed experimentally. Therefore, in terms of Raman response, the c-axis modulation is not clearly manifested.

	A_{1g}	A_{2g}	B_{1g}	B_{2g}	E_{1g}	E_{2g}	A_{1u}	A_{2u}	B_{1u}	B_{2u}	E_{1u}	E_{2u}
Γ_{total}	5	3	4	2	6	8	1	9	5	7	14	8
$\Gamma_{acoustic}$								1			1	
Γ_{optic}	5	3	4	2	6	8	1	8	5	7	13	8
Γ_{IR}								8			13	
Γ_{Raman}	5				6	8						
Γ_{silent}		3	4	2			1		5	7		8

Supplementary Table 2. Symmetry table of phonon modes of $2\times2\times1$ CDW with ISD structures. The point group is D_{6h}.

	A_g	B_{1g}	B_{2g}	B_{3g}	A_u	B_{1u}	B_{2u}	B_{3u}
Γ_{total}	14	12	12	10	8	16	17	19
$\Gamma_{acoustic}$						1	1	1
Γ_{optic}	14	12	12	10	8	15	16	18
Γ_{IR}						15	16	18
Γ_{Raman}	14	12	12	10				
Γ_{silent}					8			

Supplementary Table 3. Symmetry table of phonon modes of $2\times2\times2$ CDW with ISD structures but having interlayer π-phase shift. The point group is D_{2h}.

	A_{1g}	A_{2g}	B_{1g}	B_{2g}	E_{1g}	E_{2g}	A_{1u}	A_{2u}	B_{1u}	B_{2u}	E_{1u}	E_{2u}
Γ_{total}	12	6	9	5	15	17	2	16	9	13	25	15
$\Gamma_{acoustic}$								1			1	
Γ_{optic}	12	6	9	5	15	17	2	15	9	13	24	15
Γ_{IR}								15			24	
Γ_{Raman}	12				15	17						
Γ_{silent}	6	9	5			2		9	13		15	

Supplementary Table 4. Symmetry table of phonon modes of $2 \times 2 \times 2$ CDW with ISD and SD structures having no interlayer shift. The point group is D_{6h}.

In principle, the D_{6h} and D_{2h} point groups can be distinguished by polarization angle dependent measurements. We consider the collinear polarization configuration (denoted as XX), in which the polarizations for the incident and scattered light are kept parallel while they are co-rotated with respect to a given crystal axis. For the D_{6h} point group, the A_{1g} and E_{2g} Raman-active modes that contribute to the back-scattering response have the following

	A_{g}	$B_{1\mathrm{g}}$	$B_{2\mathrm{g}}$	$B_{3\mathrm{g}}$	A_{u}	$B_{1\mathrm{u}}$	$B_{2\mathrm{u}}$	$B_{3\mathrm{u}}$
Γ_{total}	29	23	24	20	17	31	34	38
$\Gamma_{\mathrm{acoustic}}$						1	1	1
Γ_{optic}	29	23	24	20	17	30	33	37
Γ_{IR}						30	33	37
Γ_{Raman}	29	23	24	20				
Γ_{silent}					17			

Supplementary Table 5. Symmetry table of phonon modes of $2 \times 2 \times 2$ CDW with ISD and SD structures having π-phase interlayer shift. The point group is $D_{2\mathrm{h}}$.

	$A_{1\mathrm{g}}$	$A_{2\mathrm{g}}$	$B_{1\mathrm{g}}$	$B_{2\mathrm{g}}$	$E_{1\mathrm{g}}$	$E_{2\mathrm{g}}$	$A_{1\mathrm{u}}$	$A_{2\mathrm{u}}$	$B_{1\mathrm{u}}$	$B_{2\mathrm{u}}$	$E_{1\mathrm{u}}$	$E_{2\mathrm{u}}$
Γ_{total}	26	10	20	12	35	33	6	30	16	24	45	31
$\Gamma_{\mathrm{acoustic}}$								1			1	
Γ_{optic}	26	10	20	12	35	33	6	29	16	24	44	31
Γ_{IR}								29			44	
Γ_{Raman}	26				35	33						
Γ_{silent}		10	20	12			6		16	24		31

Supplementary Table 6. Symmetry table of phonon modes of $2 \times 2 \times 4$ CDW with one ISD and three consecutive layer of SD structures having no interlayer phase shift. The point group is $D_{6\mathrm{h}}$.

Raman tensors,

$$\mathbf{R}_{A_{1\mathrm{g}}} = \begin{pmatrix} a & 0 & 0 \\ 0 & a & 0 \\ 0 & 0 & b \end{pmatrix}; \quad \mathbf{R}_{E_{2\mathrm{g}}} = \begin{pmatrix} 0 & f & 0 \\ f & 0 & 0 \\ 0 & 0 & 0 \end{pmatrix}, \begin{pmatrix} f & 0 & 0 \\ 0 & -f & 0 \\ 0 & 0 & 0 \end{pmatrix}. \tag{1}$$

The Raman scattering intensity of both modes are expected to be independent of the angle of the linear polarization θ, $I_{A_{1\mathrm{g}}}(\theta) \propto a^2$, $I_{E_{2\mathrm{g}}}(\theta) \propto f^2$. For the $D_{2\mathrm{h}}$ point group, the relevant modes have A_{g} and $B_{1\mathrm{g}}$ symmetries, and their Raman tensors are

$$\mathbf{R}_{A_{\mathrm{g}}} = \begin{pmatrix} a & 0 & 0 \\ 0 & b & 0 \\ 0 & 0 & c \end{pmatrix}; \quad \mathbf{R}_{B_{1\mathrm{g}}} = \begin{pmatrix} 0 & d & 0 \\ d & 0 & 0 \\ 0 & 0 & 0 \end{pmatrix}. \tag{2}$$

The Raman scattering intensity of both modes are expected to be anisotropic, $I_{A_{\mathrm{g}}}(\theta) \propto (a\cos^2\theta + b\sin^2\theta)^2$, $I_{B_{1\mathrm{g}}}(\theta) \propto d^2[1 - \cos(4\theta)]/2$.

Supplementary Fig. 4 shows the polarization-angle dependent data of CsV_3Sb_5 in the CDW phase (at 4 K) and the normal phase (at 100 K). We focus on the main lattice modes due to their excellent signal-to-noise ratio. Neither modes show appreciable polarization angle dependence, above and below the CDW transition. We therefore conclude that either the c-axis modulation is too weak to induce clear polarization angle dependence on the

observed Raman modes, or those candidate stacking orders with the $D_{2\mathrm{h}}$ point group (ii and iv) can be ruled out, because the $B_{1\mathrm{g}}$ mode intensity should vary sharply between zero and the maximum value, which is incompatible with the observed results.

Supplementary Note 3: Decomposition of $3M_1^+$ modes

We first calculated the character table of the three folded modes $3M_1^+$ (whose real space patterns are shown in Supplementary Figure 7) as shown in Supplementary Tab. 7. The representation of $3M_1^+$ is reducible and its decompositon onto each irreducible representation (irreps) can be done by standard textbook group theory method:

$$
\begin{aligned}
3M_1^+ &= \sum_{\oplus} a_i \Gamma_i, \\
a_i &= \frac{1}{24} \sum_{g \in D_{6\mathrm{h}}} \chi^*_{\Gamma_i}(g) \chi_{3M_1^+}(g),
\end{aligned}
$$

where $\chi_{\Gamma_i}(g)$ refers to the character of the symmetry operator g belonging to irreps Γ_i. Combining the characters of $\chi_{3M_1^+}(g)$ shown in Supplementary Tab. 7 and the irreps table of $D_{6\mathrm{h}}$, we found that the only nonzero coefficients are: $a_{A_{1\mathrm{g}}} = a_{E_{2\mathrm{g}}} = 1$.

	E	$2C_6$	$2C_3$	C_2	$3C_2'$	$3C_2''$	i	$2S_3$	$2S_6$	σ_h	$3\sigma_d$	$3\sigma_v$
$3M_1^+$	3	0	0	3	1	1	3	0	0	3	1	1

Supplementary Table 7. Character table of $3M_1^+$ under $D_{6\mathrm{h}}$.
